# Cutting the brakes and flooring the gas: how TMEPAI turns TGF-β into a tumor promoter

**DOI:** 10.18632/genesandcancer.34

**Published:** 2014-09

**Authors:** Magdalena A. Cichon, Derek C. Radisky

**Affiliations:** ^1^ Mayo Clinic Cancer Center, Jacksonville, FL, USA

**Keywords:** TGF beta, TMEPAI, breast cancer

## Abstract

In normal or nonmalignant cells, TGF-β inhibits cellular proliferation through activation of the SMAD-dependent canonical signaling pathway. Recent findings demonstrate that the protein TMEPAI1 can block the cytostatic effects of the canonical TGF-β signaling pathway, while activating cellular proliferation through the noncanonical, SMAD-independent TGF-β signaling pathway. As TMEPAI1 shows increased expression in the poor prognosis basal and HER2 intrinsic subtypes of breast cancer, these findings point to a new avenue of targeted therapy with considerable therapeutic potential.

## INTRODUCTION

**Figure 1:**
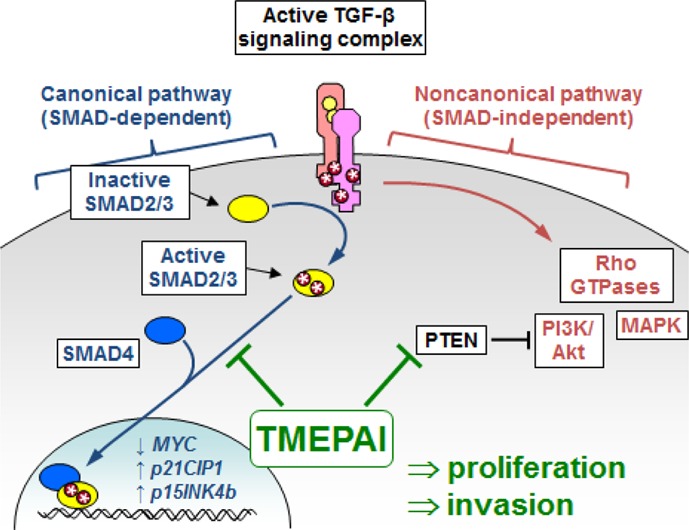
TMEPAI modulates TGF-β signaling Exposure of cells to active TGF-β leads to assembly of the tetrameric receptor complex, composed of two type I and two type II TGF-β receptor subunits (TGFBRI and TGFBRII). TGFBRII then phosphorylates TGFBRI, enabling it to activate downstream signaling pathways. In the canonical signaling pathway (left), TGFBRI phosphorylates receptor SMADs (SMAD2/3), which can then associate with SMAD4 and become translocated to the nucleus. The SMAD signaling complex can inhibit cell proliferation by inhibiting expression of *MYC* and increasing expression of *p21CIP1* and *p15INK4b*. In the noncanonical signaling pathway (right), the activated TGF-β receptor complex regulates SMAD-independent pathways, including activation of Rho family members, and can stimulate cellular proliferation through activation of MAPK and PI3K/Akt. In triple negative breast cancer cell lines, expression of TMEPAI blocks canonical signaling through sequestration of SMAD4 and potentiates the noncanonical activation of PI3K/Akt by downregulating the PI3K inhibitor PTEN.

TGF-β is a pleiotropic cytokine that has been found to modulate the function of virtually every cell type. While important developmental and normal homeostatic processes are dependent upon TGF-β, it has been primarily studied for its role in cancer development and progression, where it has been found to function both as a tumor suppressor and a tumor promoter [1]. In normal cells and in early stage tumors, TGF-β acts to block cell proliferation through the canonical signaling pathway (Fig. 1), including SMAD-dependent inhibition of MYC activation of cyclin-dependent kinase inhibitors. In more advanced tumors or in cell lines derived from these tumors, the TGF-β-dependent cytostatic effects become suppressed, and induction of the epithelial-mesenchymal transition and consequent activation of the invasive and metastatic phenotype become dominant [2, 3]. Also in advanced tumors, TGF-β can promote cell proliferation through activation of proliferative elements of the noncanonical signaling pathway (Fig. 1) [4], including activation of Akt through SMAD-independent induction of PI3K [5]. This transition from tumor-suppressive to tumor-promoting function has been described as the TGF-β paradox, and many aspects of this process remain unknown.

In 2010, a pair of publications defined key mechanisms by which the protein TMEPAI1 (transmembrane prostate androgen-induced RNA, also termed PMEPA1) could act a potent modulator of the TGF-β signaling pathway [6, 7]. TMEPAI1, itself a target gene of the canonical TGF-β signaling pathway [8], was found to inhibit the canonical TGF-β signaling through sequestration of SMAD proteins [6]. TMEPAI was also found to inhibit PTEN, leading to increased activation of PI3K/Akt by TGF-β through the noncanonical signaling pathway, and consequent promotion of cellular proliferation, including increased growth of tumors derived from human breast cancer cells orthotopically xenografted into immunocompromised mice [7]. The current publication from Singha *et al*. [9] extends these findings, showing that through coordinate regulation of the canonical and noncanonical signaling pathways (Fig. 1), TMEPAI acts to suppress the cytostatic consequences of the canonical pathway while potentiating the proliferative effects of the noncanonical pathway, thus dramatically changing the outcome of TGF-β exposure from tumor inhibition into growth promotion.

Singha *et al.* [9] employed a series of human breast cancer cell lines that lack expression of estrogen receptor (ER), progesterone receptor (PR), and HER2, and are thus designated as triple negative. Breast cancers that display the triple negative phenotype are not responsive to existing targeted therapies, and women with triple negative breast cancer (TNBC) have a particularly poor prognosis, so new approaches for treating this breast cancer subtype are urgently needed [10]. Using several different TNBC cell lines, TMEPAI was verified as activated by the canonical TGF-β pathway. This concept was reinforced by activation of TGF-β-induced expression of TMEPAI in the MDA-MB-468 TNBC cell line by re-introduction of key SMAD signaling elements. Mutagenesis experiments showed that TGF-β-induced cytostasis was due to inhibition of the canonical TGF-β signaling pathway by TMEPAI through specific sequestration of SMAD2 and SMAD3. TMEPAI was also found to activate the SMAD-independent arm of TGFβ-signaling by increasing levels of an E3 ubiquitin ligase, NEDD4, leading to degradation of PTEN and consequent activation of PI3K/Akt signaling. Further experiments showed that TMEPAI-induced loss of PTEN also resulted in increased activation of an EMT-associated transcription factor, Snail, by TGF-β, and consequent increased growth of orthotopically implanted TNBC cells. Physiological relevance for these findings was obtained through investigation of a cohort of TNBC patient biopsy samples, which showed inverse association of TMEPAI with PTEN.

**Figure 2:**
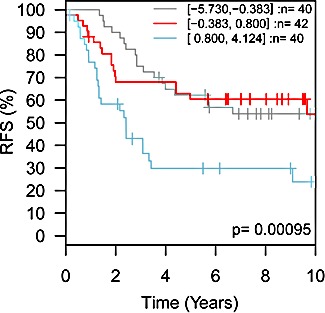
TMEPAI expression levels are prognostic for relapse-free survival in HER2^+^ breast cancer HER2-expressing intrinsic subtype breast cancer patients, divided into three groups based upon TMEPAI (PMEPA1) transcript levels (grey trace, low expression, n=40 patients; red trace, medium expression, n=42 patients; blue trace, high expression, n=40 patients) showed significant differences (p=0.00095) in relapse-free survival (RFS). Meta-analysis performed using the GOBO server (http://co.bmc.lu.se/gobo/gsa.pl).

These studies suggest that inhibition of TMEPAI may be a useful therapeutic approach for TNBC patients, although methods to accomplish this are unclear. It may be that identification and development of novel reagents to disrupt the interaction of TMEPAI with SMADs could be used in combination with PI3K- or Akt-targeting therapeutics currently in clinical trials to reactivate the cytostatic capability of TGF-β. It may be that other breast cancer subtypes could benefit from this approach as well. Interrogating a large meta-analysis of published breast cancer microarray datasets [11] in which cancers could be segregated according to PAM50-derived intrinsic subtypes [12], revealed that increased expression of TMEPAI in HER2-expressing breast cancers was associated with significantly poorer patient prognosis (Fig. 2). Strikingly, HER2 has also been shown to potentiate the protumorigenic effects of TGF-β in preclinical models [13]. Given that patients with HER2-expressing breast cancers also have a poorer relative prognosis, particularly when their cancers are initially resistant or acquire resistance to the HER2 targeting agent trastuzumab [14], investigation of the potential benefit for blocking the protumorigenic effects of TMEPAI in this cancer subtype may also be warranted.
